# Cell Density-Dependent Increase in Tyrosine-Monophosphorylated ERK2 in MDCK Cells Expressing Active Ras or Raf

**DOI:** 10.1371/journal.pone.0167940

**Published:** 2016-12-09

**Authors:** Noriyuki Kawabata, Michiyuki Matsuda

**Affiliations:** Department of Pathology and Biology of Diseases, Graduate School of Medicine, Kyoto University, Kyoto, Japan; Medical College of Wisconsin, UNITED STATES

## Abstract

The extracellular signal-regulated kinase (ERK) is one of the principal hub proteins that transmit growth signals from upstream oncogene products including Ras and BRaf to downstream effector proteins. However, there are both reports supporting and refuting the increase in ERK activity in cancer tissues expressing the active Ras and BRaf proteins. We considered that the cell density might account for this discrepancy. To examine this possibility, we prepared Madin-Darby canine kidney (MDCK) cells that expressed an active HRas, NRas, KRas, or BRaf and an ERK biosensor based on the principle of Förster resonance energy transfer (FRET). As we anticipated, expression of the active Ras or BRaf increased ERK activity at low cell densities. However, the ERK activity was markedly suppressed at high cell densities irrespective of the expression of the active Ras or BRaf. Western blotting analysis with Phos-tag gel revealed the decrease of tyrosine and threonine-diphosphorylated active ERK and the increase of tyrosine-monophosphorylated inactive ERK at high cell density. In addition, we found that calyculin A, an inhibitor for PPP-subfamily protein serine/threonine phosphatases, decreased the tyrosine-monophosphorylated ERK. Our study suggests that PPP-subfamily phosphatases may be responsible for cell density-dependent ERK dephosphorylation in cancer cells expressing active Ras or BRaf protein.

## Introduction

Ras mutation is found up to 30% of human cancer patients [1]. Raf is one of the three major effectors of Ras and is also mutated frequently in human cancers [2]. The extracellular signal-regulated kinases (ERK1/2; MAPK3/1) are considered the canonical terminus of the Ras-Raf branch, from which signals are dispatched to a number of proteins with different functions [3]. In agreement with these facts, an increase in phosphorylated active ERK (pERK) has been reported in a number of cancer tissues [4,5]. However, there are also reports claiming that pERK is not necessarily elevated in cancers harboring Ras and Raf mutations [6,7]. The failure to detect elevated pERK in Ras- or Raf-transformed cells may be ascribable to adaptation to the constitutively-active signals [8,9], or to technical pitfalls of immunohistochemistry [10]. It should also be recalled that many paradigms of oncogene signaling have been established by using rapidly-growing tissue culture cells, which may be markedly different from cancer cells in patients.

One of the marked differences between in vitro and in vivo cellular milieus is cell density. In contrast to tissue culture cells, which are often seeded at low cell densities to promote cellular replication, in vivo cancer cells grow mostly in a high cell density environment. It has been well established that inhibition of cell proliferation occurs at high cell density; this phenomenon is known as contact inhibition of cellular growth or simply contact inhibition [11,12]. In non-transformed fibroblasts [13], epithelial cells [14], and vascular endothelial cells [15], cell-to-cell contact causes downregulation of ERK and a subsequent decrease in cyclin D1. On the other hand, the loss of contact inhibition is a hallmark of cancer cells in vitro [16]. Cells infected by oncoretroviruses or transfected with oncogenes exhibit morphological changes and uncontrolled cell growth even at high cell density [17–19]. Many oncogene products exert their effect through activation of the Ras-Raf-ERK pathway; therefore, we can speculate that constitutive activation of Ras or Raf and the resulting ERK activation may contribute to the loss of contact inhibition of cancer cells. However, it has not been examined whether Ras or Raf activation is sufficient to activate ERK at high cell density.

**Fig 1:**
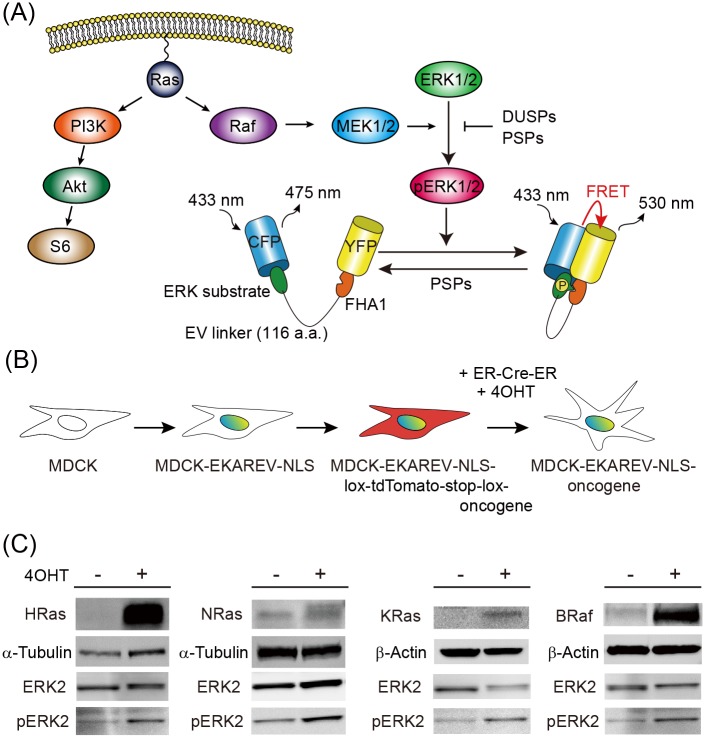
Establishment of MDCK cells expressing an active Ras or BRaf protein and a FRET biosensor for ERK activity. (A) A scheme of the Ras-Raf-MEK-ERK pathway and the mode of action of the intramolecular FRET biosensor, EKAREV-NLS. DUSPs, dual-specificity phosphatases; PSPs, protein serine/threonine phosphatases. (B) Parental MDCK cells were stably-transfected with an expression vector for EKAREV-NLS. The transfected cells were then further transfected with the pPBbsr2-lox-tdTomato-oncogene. After the introduction of ER^T2^CreER^T2^, the oncogenes were induced by 4OHT. (C) The MDCK-EKAREV-NLS-floxed-tdTomato-oncogene, the name of which is depicted in the first row, was cultured in the presence or absence of 1 μM 4OHT and analyzed by Western blotting with the antibodies listed on the left of each panel.

The development of biosensors based on Förster resonance energy transfer (FRET) has opened a path to the analysis of cellular heterogeneity and temporal changes of the activities of signaling molecules in vitro and in vivo [20,21]. For the measurement of ERK activity, we generated an intramolecular (unimolecular) FRET biosensor named EKAREV, which consists of a donor fluorescent protein CFP, an ERK substrate peptide derived from Cdc25, an optimized linker, a FHA1 phosphate binding domain, and an acceptor fluorescent protein YFP (Fig 1A) [22,23]. Activated ERK phosphorylates the substrate peptide and induces intramolecular binding of the FHA1 domain to the phosphorylated peptide, thereby bringing the two fluorescent proteins in close proximity to evoke FRET. The FRET biosensor is reversed to the pre-phosphorylation state by protein serine/threonine phosphatases (PSPs). The halflife of active ERK is approximately 30 seconds, which is slow enough to be monitored by the FRET biosensors [24]. Thus, by measuring the fluorescence intensities derived from FRET and CFP (FRET/CFP ratio for brevity), we can obtain spatiotemporal information of the activity balance between ERK and PSPs in living cells.

The EKAREV FRET biosensor has already been used in many studies. For example, we and others have found that ERK can be stochastically activated in tissue culture cells [25,26], and such stochastic ERK activation can be propagated to neighboring cells both in vitro and in vivo [25,27]. Here, by using EKAREV, we examined the effects of oncogenic Ras and BRaf proteins on ERK activity in Madin-Darby canine kidney (MDCK) cells cultured at high cell density. We found that, at high cell density, ERK activity was markedly decreased even in the Ras- or BRaf-expressing MDCK cells. This suppression appears to be caused by the activation of PSPs and resulting accumulation of inactive tyrosine-monophosphorylated ERK. Our observations imply that the discrepancy between the expression of active Ras or BRaf and ERK activity in cancer tissues might reflect the difference in cell density-dependent activity of PSPs.

## Materials and Methods

### Plasmids and retroviruses

The nuclear FRET biosensor for ERK, EKAREV-NLS, was prepared as reported previously [23]. Either Tol2 or piggyBac transposase was used for the stable expression of genes in MDCK cells [28,29]. The coding sequence of EKAREV-NLS was inserted into pT2AL200R175-CAGGS [29] to generate pT2A-3905NLS (pT2A-EKAREV-NLS). pCAGGS-T2TP is an expression vector for the Tol2 transposase. pPBbsr2-lox-tdTomato-KRasV12 was based on pPBbsr2 [25,28] and comprised of a floxed tdTomato gene [30], a stop codon, and a human KRasG12V gene in this order. pPBbsr2-lox-tdTomato-NRasV12, HRasV12, and BRafV600E were generated by the standard PCR-based technology. pCMV-mPBase (neo-) is an expression vector for piggyBac transposase. pCX4puro-ER^T2^CreER^T2^ is derived from the pCX4puro retroviral expression plasmid [31] and comprised of ER^T2^CreER^T2^ [32]. The retroviral packaging plasmid pGP and the envelope plasmid pCMV-VSV-G-RSV-Rev were used to generate recombinant retroviruses as described previously [33].

### Establishment of MDCK cells expressing a FRET biosensor

MDCK cells were purchased from the RIKEN BioResource Center (no. RCB0995), and maintained in MEM (no. 51200038; Life Technologies, Carlsbad, CA) supplemented with 10% FBS (Equitech-Bio, Woburn, MA), 1% non-essential amino acids (no. 11140050; Life Technology), 1% GlutaMAX Supplement (no. 35050061; Life Technology) and 1 mm sodium pyruvate (no. 11360070; Life Technology) in a 5% CO_2_ humidified incubator at 37°C. To obtain MDCK-EKAREV-NLS cells, MDCK cells were co-transfected with pT2A-EKAREV-NLS and pCAGGS-T2TP and sorted by FACS as previously described [33]. MDCK-EKAREV-NLS cells were co-transfected with pCMV-mPBase(neo-) and pPBbsr2-lox-tdTomato-HRasV12, NRasV12, KRasV12, or -BrasV600E and selected by blasticidin S (no. 203350; Calbiochem, San Diego, CA). These cells were infected with the retrovirus carrying the ER^T2^CreER^T2^ gene, and selected by puromycin and sorted by FACS. After single cell cloning, the oncogenes were induced by 1 μM tetrahydroxytamoxifen (4OHT; no. H7904; Sigma-Aldrich). After two-day incubation, tdTomato-negative cells were used for further analyses.

### Reagents and antibodies

Calyculin A (no. C5552) was purchased from Sigma-Aldrich (St. Louis, MO). Okadaic acid was from Calbiochem (no. 459620). Phos-tag acrylamide AAL-107 (no. 304–93521) was purchased from Wako Pure Chemical Industries, Ltd. (Osaka, Japan). The following antibodies were purchased from Cell Signaling Technology (Danvers, MA): Anti-p44/42 MAPK (Erk1/2) rabbit antibody (no. 4695), anti-phospho-p44/42 MAPK (Erk1/2) mouse antibody (no. 9106S), anti-S6 ribosomal protein rabbit antibody (no. 2317S), anti-phospho-S6 ribosomal protein (Ser235/236) rabbit antibody (no. 4858S), anti-phospho-Akt (Ser473) mouse antibody (no. 4060), anti-phospho-Akt (Thr308) rabbit antibody (no. 13038S), anti-pan Akt rabbit antibody (no. 4691S) and anti-β-actin rabbit antibody (no. 4790S). Anti-α-tubulin mouse antibody was from Calbiochem (no. CP06). Odyssey Blocking Buffer (TBS) (no. 927–50000; LI-COR Biosciences), IRDye680LT goat anti-rabbit IgG antibody (no. 925–68021) and IRDye800CW donkey anti-mouse IgG antibody (no. 925–32212) were obtained from LI-COR Bioscience (Lincoln, NE).

### Polyacrylamide gel electrophoresis and Western blotting

Western blotting and Phos-tag polyacrylamide gel electrophoresis were performed essentially as described previously [34]. Briefly, cells were lysed in SDS sample buffer [62.5 mM Tris-HCl (pH 6.8), 12% glycerol, 2% SDS, 0.004% bromophenol blue and 5% 2-mercaptoethanol] at a concentration of 430 cells/μL. After sonication and boiling at 95°C, the samples were separated by SDS-PAGE on SuperSep Ace 5–20% pre-cast gels (no. 197–15011; Wako Pure Chemical). For Phos-tag Western blottings of ERK, 5.0 × 10^−5^ M Phos-tag Acrylamide (no. 304–93521; Wako Pure Chemical) and 1.0 × 10^−4^ M MnCl_2_ (no. 21211; Nacalai tesque, Tokyo, Japan) were added to conventional SDS-polyacrylamide separation gels according to the manufacturer’s protocol. The samples were then transferred to PVDF membranes (no. IPFL00010; Merck Millipore, Darmstadt, Germany). After 60-min of incubation with Odyssey blocking buffer (TBS) at room temperature, the membranes were incubated overnight at 4°C with primary antibodies that were 1,000-fold diluted in Can Get Signal Immunoreaction Enhancer Solution 1 (no. NKB-201; TOYOBO, Osaka, Japan). The primary antibodies were visualized with fluorescent Dye-tagged secondary antibodies that were 20,000-fold diluted in Odyssey blocking buffer (TBS) and an Odyssey Infrared Imaging System (LI-COR).

### Imaging

Live cell imaging was performed basically as previously reported [23,35]. In general, images of MDCK cells expressing FRET biosensors were acquired every 5 to 10 min. After 30 min of imaging under the pre-stimulation conditions, the cells were treated with reagents and further imaged for 60 min. For the wound-healing assay, Culture-Insert 2 Well^™^ (no. 81176; ibidi, Martinsried, Germany) was used. Imaging was performed with an inverted microscope (IX83; Olympus, Tokyo, Japan) equipped with 10× (NA 0.40), 20× (NA 0.75) and 40× (NA 0.95) objective lenses (UPlan SApo; Olympus), a cooled CCD camera (Cool SNAP-K4; Roper Scientific), an LED illumination system (CoolLED precisExcite; Molecular Devices), an IX2-ZDC laser-based autofocusing system (Olympus) and a D-XY30100T-Meta automatically programmable XY stage (SIGMA KOKI, Tokyo, Japan). The following filters used for the dual emission imaging studies were obtained from Omega Optical (Brattleboro, VT): an XF1071 (440AF21) excitation filter, an XF2034 (455DRLP) dichroic mirror, and three emission filters (FF01-483/32-25 for CFP, FF01-542/27-25 for FRET and YFP, and FF01-593/40-25 for tdTomato).

### Image processing

Metamorph software (Molecular Devices, Sunnyvale, CA) was used for background noise subtraction and image analysis. Background intensities were determined by using an empty culture dish with the same amount of media. After background subtraction, the FRET/CFP ratio images were represented in the intensity-modulated display (IMD) mode. In the IMD mode, eight colors from red to blue are used to represent the FRET/CFP ratio, with the intensity of each color indicating the mean intensity of FRET and CFP channels. Heatmap images representing the relationship between the FRET/CFP ratio and time course were generated using MATLAB software (MathWorks, Natick, MA).

### Statistics

Statistical analyses were conducted using R software (ver. 3.1.3). In the beeswarm plot, a bold line indicates the median, and fine lines, if any, denote the upper and lower quantiles, respectively. To compare two sets of data, Student’s t test or Welch’s t test was performed according to a result of F test. As a multiple comparison test, Tukey’s honestly significant difference test or Steel-Dwass test was used after validation of homoscedasticity by Bartlett’s test. In all tests, values of p < 0.05 were considered significant.

## Results

### Establishment of MDCK cells expressing an active Ras or BRaf protein and a FRET biosensor for ERK activity

For the measurement of ERK activity in living cells, a FRET biosensor for ERK, EKAREV-NLS, was expressed in the nucleus of MDCK cells. The 4OHT-inducible Cre-dependent expression system was employed to minimize the inter-clonal variation (Fig 1A and 1B). Active mutants of four oncogene products, HRasG12V, NRasG12V, KRasG12V, and BRafV600E, were designed to be expressed in MDCK cells. Induction of each oncogene product was indeed confirmed by Western blotting analysis (Fig 1C).

### Cell density-dependent suppression of ERK activity in MDCK cells expressing an active Ras or BRaf protein

**Fig 2:**
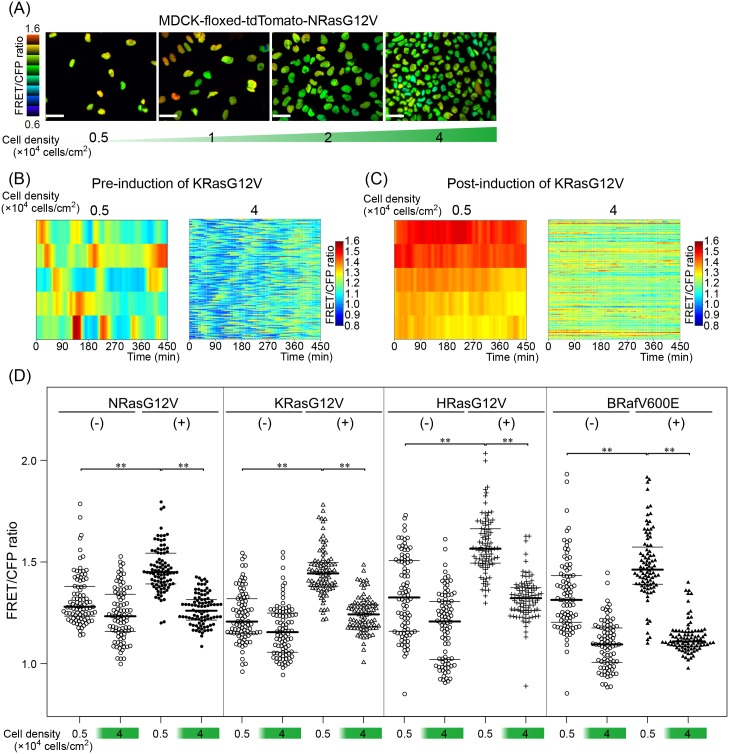
Cell density-dependent suppression of ERK activity in MDCK cells expressing an active Ras or BRaf. (A) MDCK-floxed-tdTomato-NRasG12V cells before 4OHT induction were plated at the cell densities depicted at the bottom of each panel and imaged with a fluorescence microscope. FRET/CFP ratio images in the intensity-modulated display mode indicate the ERK activity. Bars, 50 μm. (B) MDCK-floxed-tdTomato-KRasG12V cells were time-lapse imaged for 450 min. The mean FRET/CFP ratios at low cell density (0.5×10^4^ cells/cm^2^) and at high cell density (4×10^4^ cells/cm^2^) are shown by heatmaps. Each row represents time course of FRET/CFP ratio of single cell. The numbers of analyzed cells are 5 for low cell density and 150 for high cell density. (C) Similar experiments were performed after 4OHT induction. (D) MDCK-floxed-tdTomato-oncogene cells before and after 4OHT induction were plated at low and high cell densities and imaged with a fluorescent microscope. The FRET/CFP ratios of 90 cells from three independent experiments are plotted for each condition.

To examine the effect of the cell density on ERK activity, we first plated the MDCK-EKAREV-NLS cells without induction of oncogene at increasing cell densities. In FRET/CFP ratio images acquired before induction of NRasG12V, as reported previously [25,26], ERK activity was heterogeneous at low cell density (Fig 2A). A timelapse video and a heatmap of other pre-induction cell line, MDCK-EKAREV-NLS-floxed-tdTomato-KRasG12V cells, also showed this heterogeneity and it was generated by stochastic ERK activity pulses (Fig 2B left). However, at high cell density, both the basal ERK activity and the stochastic ERK activity pulse were suppressed (Fig 2B right, S1 Movie). We next induced KRasG12V by 4OHT and analyzed their activities at low and high cell densities (Fig 2C, S2 Movie). At low cell density, KRasG12V cells induced robust ERK activation and abolished ERK activity pulses (Fig 2C left). However, ERK activity was markedly suppressed to a level comparable to that of the pre-induced cells at high cell density (Fig 2C right). We also analyzed other MDCK cell lines that encoded HRasG12V, NRasG12V, and BRafV600E about ERK activity at different cell densities and the expression of oncogenic mutants. Again, the cells were plated at low and high cell densities and examined for ERK activity (Fig 2D). The beeswarm plots confirmed that the induction of oncogene products raised the ERK activity at low cell density, but that the oncogene products-induced high ERK activation could be antagonized by high cell density. These observations clearly indicate that the high ERK activity induced by active oncogene products is subject to the suppression by high cell density.

### ERK activity change in the wound-healing assay

**Fig 3:**
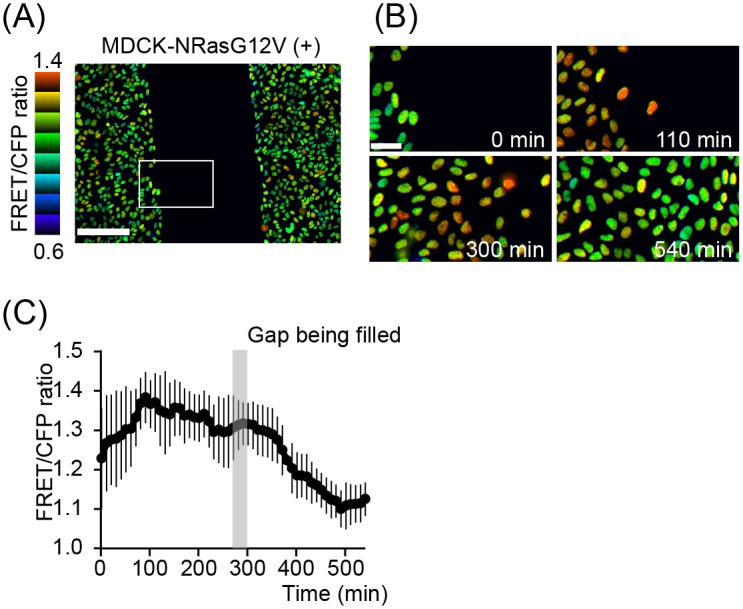
ERK activity change in the wound-healing assay. (A) MDCK cells expressing NRasG12V and EKAREV-NLS were plated in Culture-Insert 2 Well (ibidi) at high cell density and imaged with a fluorescence microscope. No difference in ERK activity was detected between cells in contact with and cells apart from the silicon insert. Bar, 200 μm. (B and C) The wound-healing assay was started by removing the silicon insert. Representative consecutive FRET/CFP ratio images. Bar, 50 μm (B). The FRET/CFP ratios in 14 cells at the border were monitored and plotted over time. Mean ± SD (C).

The low ERK activity at high cell density suggests the two following possibilities. First, low cell density, i.e., a large cell-to-substratum area, may be required for the induction of high ERK activity by oncogene products. Second, a free edge, i.e., a plasma membrane that does not face the other cells, may be required for ERK activation by the oncogene products. In support of the latter view, it has been proposed that Ras is activated at the free edge but not at the plasma membrane facing the neighboring cells [36]. To resolve this question, we performed a wound healing assay. MDCK cells expressing NRasG12V were plated on two chambers separated by a silicon wall, which could be removed during the experiment. No difference in ERK activity was found whether or not the cells were facing the silicon wall (Fig 3A). Upon removal of the silicon wall, the cells that had faced the silicon wall moved forward and exhibited a rapid increase in ERK activity, even though these cells were still in contact with the follower cells (Fig 3B and 3C). When the cells of both edges filled the gap, ERK activity was decreased gradually. In the leading cells that faced to the silicon insert, the length of the cell border in contact with the other cells did not change significantly during cell migration. Therefore, the high cell density, but not the cell-to-cell contact, appears to suppress ERK activity in MDCK-NRasG12V cells cultured at high cell density.

### Accumulation of tyrosine-monophosphorylated inactive ERK at high cell density

**Fig 4:**
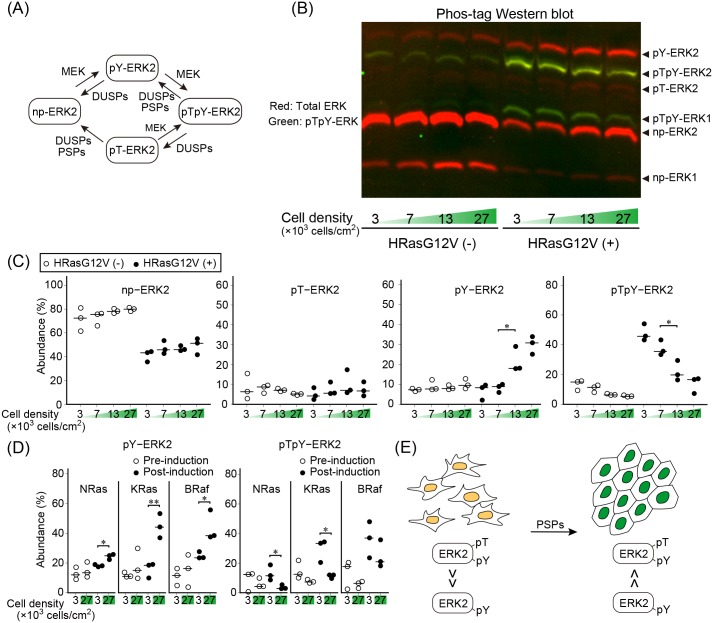
Increase in tyrosine-monophosphorylated ERK at high cell density. (A) A schematic representation of the ERK phosphorylation status. np-ERK2, non-phosphorylated ERK2; pT-ERK2, ERK2 monophosphorylated on Thr^185^; pY-ERK2, ERK2 monophosphorylated on Tyr^187^; pTpY-ERK2, ERK2 diphosphorylated on Thr^185^ and Tyr^187^; DUSPs, dual specificity phosphatases; PSPs, protein serine/threonine phosphatases. (B) MDCK-floxed-tdTomato-HRasG12V cells before and after 4OHT induction were plated at the cell densities depicted at the bottom of each lane and analyzed by Western blotting analysis with Phos-tag gels. The filters were probed with anti-ERK and anti-phospho-ERK (Thr^185^/Tyr^187^) antibodies. The yellow bands indicate pTpY-ERK1 and pTpY-ERK2. (C) The fluorescence intensities of ERK2 proteins were quantified and the percentage of each phospho-isoform is plotted. Data obtained from three independent experiments are shown. *p < 0.05. (D) Similar experiments were performed with MDCK cells before and after the induction of NRasG12V, KRasG12V, or BRafV600E as indicated. Data are shown only for pY-ERK2 and pTpY-ERK2 for simplicity. *p < 0.05; **p < 0.01. (E) A schematic of the mechanism of decrease in pTpY-ERK2 at high cell density.

How can ERK activity be suppressed in MDCK cells expressing the active Ras or BRaf? To answer this question, we examined the phosphorylation status of ERK by using Phos-tag gels as described previously [34]. ERK has four distinct forms according to the phosphorylation status of tyrosine and threonine residues within the catalytic loop (Fig 4A). Non-phosphorylated ERK (np-ERK) is first phosphorylated on tyrosine to generate tyrosine-monophosphorylated ERK (pY-ERK). pY-ERK is then further phosphorylated to generate tyrosine and threonine diphosphorylated ERK (pTpY-ERK), which is the enzymatically-active form. Dephosphorylation can follow two pathways via either pY-ERK or threonine-monophosphorylated ERK (pT-ERK). These four states of ERK could be separated by Phos-tag gels and probed with an anti-ERK antibody (Fig 4B). Because ERK2 is significantly more abundant than ERK1, we concentrated our efforts on quantification of the amount of ERK2 phospho-isoforms in MDCK cells with or without HRasG12V expression (Fig 4C). As was anticipated from the imaging data, the amount of pTpY-ERK was increased by the induction of HRasG12V and correlated inversely with the cell density. Notably, this decrease in pTpY-ERK at high cell density was accompanied by the increase in pY-ERK. Meanwhile, np-ERK was not significantly changed by the difference of cell density. Similar results were obtained by using MDCK cells expressing NRasG12V, KRasG12V, or BRafV600E (Fig 4D). Taken together, our observations raised the possibility that serine/threonine phosphatases were activated to invoke the shift from pTpY-ERK2 to pY-ERK2 under the high cell density condition (Fig 4E).

Recently, Iwamoto et al. reported that pTpY-ERK and pT-ERK, but not pY-ERK, are increased in the Ras-transformed keratinocyte cell line HaCaT A5 [37]. In this cell line, DUSP6 has been shown to contribute to the accumulation of pT-ERK. The cause of the discrepancy may be the difference of cell type.

### Decreased phosphorylation of Akt and S6 ribosomal protein at high cell density

**Fig 5:**
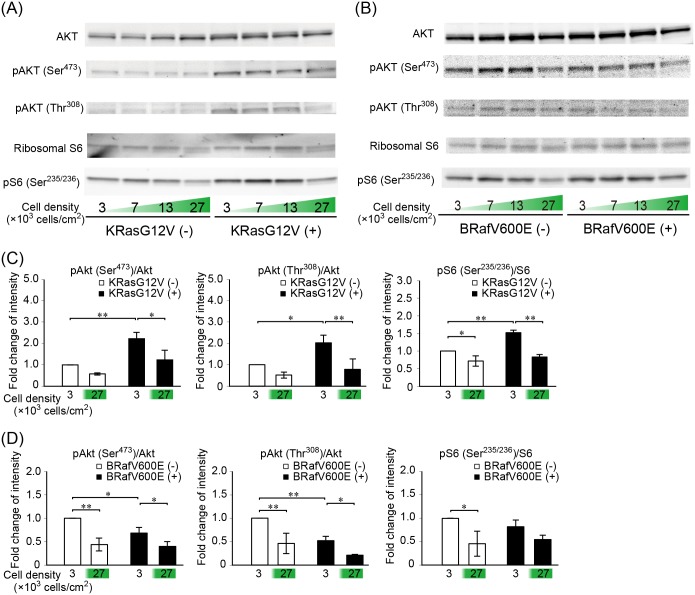
Cell density-dependent decrease of phosphorylation of Akt and S6 ribosomal protein. (A and B) MDCK cells before and after KRasG12V or BRafV600E induction were plated at the cell densities depicted at the bottom of each column and analyzed by Western blotting with anti-Akt, anti-phospho-Akt (Thr^308^), anti-phospho-Akt (Ser^473^), anti-S6 ribosomal protein antibody, and anti-phospho-S6 ribosomal protein (Ser^235/236^). (C and D) Quantification of each band in the blotting. Y-axis represents fold change comparing with the pre-induction cells at the low cell density as control.

If serine/threonine phosphatase activity was increased at high cell density, decreased phosphorylation would be observed not only in ERK2 but also other downstream effector proteins of Ras. To examine whether this is the case, the phosphorylations of Akt and its downstream effector S6 ribosomal protein were examined by Western blotting (Fig 5). Upon induction of KRasG12V, phosphorylations of Akt and the S6 protein were increased at low cell density. This increase in phosphorylation was attenuated at high cell density (A and C). Upon the induction of BRafV600E, neither phosphorylation of Akt nor that of the S6 ribosomal protein was increased, which may suggest negative regulation at the level of Ras (B and D). Nevertheless, the phosphorylations of Akt and the S6 ribosomal protein were decreased at high cell density, implying that serine/threonine phosphatase activity was increased at high cell density.

### Decreased pY-ERK2 in calyculin A-treated MDCK cells at high cell density

To gain further insight into the nature of phosphatases upregulated in confluent MDCK cells, we used inhibitors of PSPs. PSPs comprise three major families: phosphoprotein phosphatases (PPPs), metal-dependent protein phosphatases (PPMs), and the aspartate-based phosphatases [38,39]. The largest PPP-family proteins include PP1 and PP2A, which are ubiquitously expressed. Calyculin A inhibits a broad range of both PP2A and PP1. Okadaic acid inhibits PP2A completely, but inhibits PP1 only partially at the concentrations used in this study [40,41]. Neither of them inhibits PPMs or the aspartate-based phosphatases [42].

**Fig 6:**
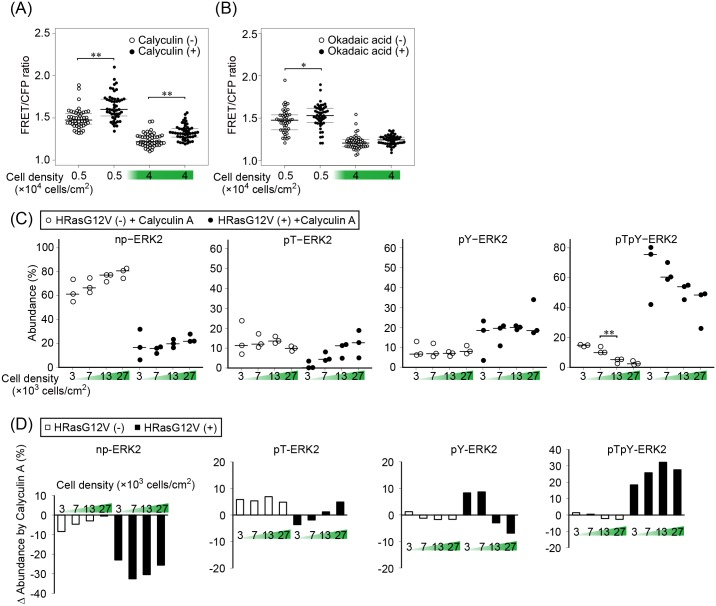
Calyculin A-induced decrease of tyrosine-monophosphorylated ERK2 at high cell density. (A and B) MDCK cells were stimulated with 5 nM calyculin A (A) or 500 nM okadaic acid (B). The mean and upper/lower quantiles of FRET/CFP ratios are plotted before and after 30 min incubation, respectively (n = 50). *p < 0.05; **p < 0.01. (C) Cells were plated at the indicated cell density, cultured overnight, and incubated with 5 nM calyculin A for 30 minutes, followed by Phos-tag Western blot analysis with anti-ERK and anti-phospho-ERK (Thr^185^/Tyr^187^) antibodies as shown in Fig 4. (D) The difference between the fractions of calyculin A-treated cells (C) and non-treated cells (Fig 4C) is shown.

MCDK-HRasG12V cells plated at low and high cell densities were treated with calyculin A and okadaic acid and examined for ERK activity by FRET imaging (Fig 6A and 6B). Calyculin A, but not okadaic acid, was found to increase the ERK activity, suggesting that PP1 is responsible for the confluency-dependent suppression of ERK. To further confirm the effect of calyculin A, the phosphorylation status of ERK was examined by Western blotting with Phos-tag gels. In agreement with the imaging data, calyculin A was found to decrease np-ERK2 and to increase pTpY-ERK2 at all cell densities (Fig 6C and 6D). Importantly, calyculin A decreased pY-ERK2 at high cell density in MDCK cells expressing HRasG12V, which supports the findings shown in Fig 3 that accumulation of pY-ERK was the primary cause of ERK suppression at high cell density.

## Discussion

The loss of contact inhibition is a hallmark of cancer cells in vitro [19]. In earlier studies, expression of active oncogenes was believed to be sufficient to induce contact inhibition; however, later studies have shown that additional mutations endow robust malignant features [43,44]. In agreement, we have also found that expression of Ras or BRaf was not sufficient to override the cell density-dependent suppression of ERK2 activity. We have further shown that the suppression of ERK activity was caused primarily by the transition of the active pTpY-ERK2 to inactive pY-ERK2. The sensitivity to calyculin A, but not to okadaic acid, suggests the involvement of PP1 in the cell density-dependent suppression of ERK2.

Dephosphorylation is the reverse reaction of protein phosphorylation and should play important roles in the regulation of cancer cell growth. Nevertheless, the number of PSPs are markedly less than that of protein kinases, implying that PSPs revert broad spectrum of protein kinase reactions, each of which is dictated by a specific protein kinase(s) [39]. In contrast to the pro-oncogenic roles played by protein kinases, PSPs are generally regarded as the tumor suppressor. For example, PP2A is known to regulate cell cycle and apoptosis of cancer cells. Furthermore, recent preclinical studies have shown that PP2A-activating drugs can antagonize cancer development and progression [45]. Similarly to PP2A, PP1 regulates a number of biological phenomena including cell division, apoptosis, metabolism, protein synthesis, regulation of cytoskeleton and ion receptors [46]. Due to the pleiotropic functions of PP1, there are some discrepancies on the mechanism by which PP1 regulates ERK. PP1 dephosphorylates phospho-Ser^259^ of CRaf, which binds to 14-3-3 and thereby suppresses kinase activity. Consequently, PP1 activates ERK via CRaf activation [47,48]. On the other hand, PP1 has been shown to negatively control ERK via DARPP-32 protein in a subset of medium-size spiny neurons of the dorsal striatum and nucleus accumbens [49]. We suggest that PP1 negatively regulates ERK activity via dephosphorylation of threonine (Fig 4) in a cell density dependent manner. Therefore, the action of PP1 on the Ras-Raf-ERK pathway appears to be dependent on not only the cell types but also the cell density. It should also be mentioned that the discrepancy between the expression of active Ras or BRaf and ERK activity in cancer tissues may be ascribable to the activity of PP1 in each cancer tissue.

## Supporting Information

S1 Movie Pre-induction of KRasG12V. (MOV)

S2 Movie Post-induction of KRasG12V. (MOV)

## References

[1] Bos JL. ras oncogene in human cancer: a review. Cancer Res. 1989; 49: 4682–4689. 2547513

[2] Garnett MJ, Marais R. Guilty as charged: B-RAF is a human oncogene. Cancer Cell. 2004; 6: 313–319. 10.1016/j.ccr.2004.09.022 15488754

[3] Nishida E, Gotoh Y. The MAP kinase cascade is essential for diverse signal transduction pathways. Trends Biochem Sci. 1993; 18: 128–131. 8388132 10.1016/0968-0004(93)90019-j

[4] Sebolt-Leopold JS, Herrera R. Targeting the mitogen-activated protein kinase cascade to treat cancer. Nat Rev Cancer. 2004; 4: 937–947. 10.1038/nrc1503 15573115

[5] Schubbert S, Shannon K, Bollag G. Hyperactive Ras in developmental disorders and cancer. Nat Rev Cancer. 2007; 7: 295–308. 10.1038/nrc2109 17384584

[6] Houben R, Vetter-Kauczok CS, Ortmann S, Rapp UR, Broecker EB, Becker JC. Phospho-ERK staining is a poor indicator of the mutational status of BRAF and NRAS in human melanoma. J Invest Dermatol. 2008; 128: 2003–2012. 10.1038/jid.2008.30 18323787

[7] Smalley KS, Contractor R, Haass NK, Lee JT, Nathanson KL, Medina CA, et al Ki67 expression levels are a better marker of reduced melanoma growth following MEK inhibitor treatment than phospho-ERK levels. Br J Cancer. 2007; 96: 445–449. 10.1038/sj.bjc.6603596 17245336 PMC2360037

[8] Kholodenko BN. Negative feedback and ultrasensitivity can bring about oscillations in the mitogen-activated protein kinase cascades. Eur J Biochem. 2000; 267: 1583–1588. 10712587 10.1046/j.1432-1327.2000.01197.x

[9] Ahmed S, Grant KG, Edwards LE, Rahman A, Cirit M, Goshe MB, et al Data-driven modeling reconciles kinetics of ERK phosphorylation, localization, and activity states. Mol Syst Biol. 2014; 10: 718 10.1002/msb.134708 24489118 PMC4023404

[10] Mandell JW. Immunohistochemical assessment of protein phosphorylation state: the dream and the reality. Histochem Cell Biol. 2008; 130: 465–471. 10.1007/s00418-008-0474-z 18648845 PMC2522329

[11] Eagle H, Levine EM. Growth regulatory effects of cellular interaction. Nature. 1967; 213: 1102–1106. 6029791 10.1038/2131102a0

[12] Abercrombie M. Contact inhibition and malignancy. Nature. 1979; 281: 259–262. 551275 10.1038/281259a0

[13] Wayne J, Sielski J, Rizvi A, Georges K, Hutter D. ERK regulation upon contact inhibition in fibroblasts. Mol Cell Biochem. 2006; 286: 181–189. 10.1007/s11010-005-9089-z 16467968

[14] Li S, Gerrard ER Jr., Balkovetz DF. Evidence for ERK1/2 phosphorylation controlling contact inhibition of proliferation in Madin-Darby canine kidney epithelial cells. Am J Physiol Cell Physiol. 2004; 287: C432–439. 10.1152/ajpcell.00020.2004 15070810

[15] Vinals F, Pouyssegur J. Confluence of vascular endothelial cells induces cell cycle exit by inhibiting p42/p44 mitogen-activated protein kinase activity. Mol Cell Biol. 1999; 19: 2763–2772. 10082542 10.1128/mcb.19.4.2763 PMC84069

[16] Hanahan D, Weinberg RA. Hallmarks of cancer: the next generation. Cell. 2011; 144: 646–674. 10.1016/j.cell.2011.02.013 21376230

[17] Pastan I, Willingham M. Cellular transformation and the 'morphologic phenotype' of transformed cells. Nature. 1978; 274: 645–650. 209337 10.1038/274645a0

[18] Cooper GM. Cellular transforming genes. Science. 1982; 217: 801–806. 6285471 10.1126/science.6285471

[19] McCoy MS, Toole JJ, Cunningham JM, Chang EH, Lowy DR, Weinberg RA. Characterization of a human colon/lung carcinoma oncogene. Nature (London). 1983; 302: 79–81. 6298638 10.1038/302079a0

[20] Oldach L, Zhang J. Genetically encoded fluorescent biosensors for live-cell visualization of protein phosphorylation. Chem Biol. 2014; 21: 186–197. 10.1016/j.chembiol.2013.12.012 24485761 PMC4050661

[21] Miyawaki A, Niino Y. Molecular spies for bioimaging--fluorescent protein-based probes. Mol Cell. 2015; 58: 632–643. 10.1016/j.molcel.2015.03.002 26000848

[22] Harvey CD, Ehrhardt AG, Cellurale C, Zhong H, Yasuda R, Davis RJ, et al A genetically encoded fluorescent sensor of ERK activity. Proc Natl Acad Sci U S A. 2008; 105: 19263–19268. 10.1073/pnas.0804598105 PMC2614750 19033456

[23] Komatsu N, Aoki K, Yamada M, Yukinaga H, Fujita Y, Kamioka Y, et al Development of an optimized backbone of FRET biosensors for kinases and GTPases. Mol Biol Cell. 2011; 22: 4647–4656. 10.1091/mbc.E11-01-0072 21976697 PMC3226481

[24] Fujioka A, Terai K, Itoh RE, Aoki K, Nakamura T, Kuroda S, et al Dynamics of the Ras/ERK MAPK cascade as monitored by fluorescent probes. J Biol Chem. 2006; 281: 8917–8926. 10.1074/jbc.M509344200 16418172

[25] Aoki K, Kumagai Y, Sakurai A, Komatsu N, Fujita Y, Shionyu C, et al Stochastic ERK activation induced by noise and cell-to-cell propagation regulates cell density-dependent proliferation. Mol Cell. 2013; 52: 529–540. 10.1016/j.molcel.2013.09.015 24140422

[26] Albeck JG, Mills GB, Brugge JS. Frequency-Modulated Pulses of ERK Activity Transmit Quantitative Proliferation Signals. Mol Cell. 2013; 19: 249–261. 10.1016/j.molcel.2012.11.002 PMC4151532 23219535

[27] Hiratsuka T, Fujita Y, Naoki H, Aoki K, Kamioka Y, Matsuda M. Intercellular propagation of extracellular signal-regulated kinase activation revealed by in vivo imaging of mouse skin. Elife. 2015; 4: e05178 10.7554/eLife.05178 25668746 PMC4337632

[28] Yusa K, Rad R, Takeda J, Bradley A. Generation of transgene-free induced pluripotent mouse stem cells by the piggyBac transposon. Nature Methods. 2009; 6: 363–369. 10.1038/nmeth.1323 19337237 PMC2677165

[29] Urasaki A, Morvan G, Kawakami K. Functional dissection of the Tol2 transposable element identified the minimal cis-sequence and a highly repetitive sequence in the subterminal region essential for transposition. Genetics. 2006; 174: 639–649. 10.1534/genetics.106.060244 16959904 PMC1602067

[30] Shaner NC, Campbell RE, Steinbach PA, Giepmans BN, Palmer AE, Tsien RY. Improved monomeric red, orange and yellow fluorescent proteins derived from Discosoma sp. red fluorescent protein. Nat Biotechnol. 2004; 22: 1567–1572. 10.1038/nbt1037 15558047

[31] Akagi T, Sasai K, Hanafusa H. Refractory nature of normal human diploid fibroblasts with respect to oncogene-mediated transformation. Proc Natl Acad Sci U S A. 2003; 100: 13567–13572. 10.1073/pnas.1834876100 14597713 PMC263854

[32] Matsuda T, Cepko CL. Controlled expression of transgenes introduced by in vivo electroporation. Proc Natl Acad Sci U S A. 2007; 104: 1027–1032. 10.1073/pnas.0610155104 17209010 PMC1764220

[33] Sakurai A, Matsuda M, Kiyokawa E. Activated ras protein accelerates cell cycle progression to perturb madin-darby canine kidney cystogenesis. J Biol Chem. 2012; 287: 31703–31711. 10.1074/jbc.M112.377804 22829590 PMC3442505

[34] Aoki K, Yamada M, Kunida K, Yasuda S, Matsuda M. Processive phosphorylation of ERK MAP kinase in mammalian cells. Proc Natl Acad Sci U S A. 2011; 108: 12675–12680. 10.1073/pnas.1104030108 21768338 PMC3150946

[35] Aoki K, Matsuda M. Visualization of small GTPase activity with fluorescence resonance energy transfer-based biosensors. Nature Protocol. 2009; 4: 1623–1631. 10.1038/nprot.2009.175 19834477

[36] Mochizuki N, Yamashita S, Kurokawa K, Ohba Y, Nagai T, Miyawaki A, et al Spacio-temporal images of growth factor-induced activation of Ras and Rap1. Nature (London). 2001; 411: 1065–1068. 11429608 10.1038/35082594

[37] Iwamoto N, D’Alessandro LA, Depner S, Hahn B, Kramer BA, Lucarelli P, et al Context-specific flow through the MEK/ERK module produces cell- and ligand-specific patterns of ERK single and double phosphorylation. Science Signaling. 2016; 9: ra13–ra13. 10.1126/scisignal.aab1967 26838549

[38] Brautigan DL. Protein Ser/Thr phosphatases--the ugly ducklings of cell signalling. The FEBS J. 2013; 280: 324–345. 10.1111/j.1742-4658.2012.08609.x 22519956

[39] Shi Y. Serine/threonine phosphatases: mechanism through structure. Cell. 2009; 139: 468–484. 10.1016/j.cell.2009.10.006 19879837

[40] Cohen P, Klumpp S, Scelling DL. An improved procedure for identifying and quantitating protein phosphatases in mammalian tissues. FEBS Lett. 1989; 250: 596–600. 2546812 10.1016/0014-5793(89)80803-8

[41] Ishihara H, Martin BL, Brautigan DL, Karaki H, Ozaki H, Kato Y, et al Calyculin A and okadaic acid: inhibitors of protein phosphatase activity. Biochem Biophys Res Commun. 1989; 159: 871–877. 2539153 10.1016/0006-291x(89)92189-x

[42] Swingle M, Ni L, Honkanen RE. Small Molecule Inhibitors of Ser/thr Protein Phosphatases: Specificity, Use and Common Forms of Abuse. Methods Mol Biol (Clifton, NJ). 2007; 365: 23–38. 10.1385/1-59745-267-X:23 PMC2709456 17200551

[43] Kinzler KW, Vogelstein B. Lessons from Hereditary Colorectal Cancer. Cell. 1996; 87: 159–170. 8861899 10.1016/s0092-8674(00)81333-1

[44] Burd CE, Liu W, Huynh M V, Waqas MA, Gillahan JE, Clark KS, et al Mutation-specific RAS oncogenicity explains NRAS codon 61 selection in melanoma. Cancer Discov. 2014; 4: 1418–1429. 10.1158/2159-8290.CD-14-0729 25252692 PMC4258185

[45] Perrotti D, Neviani P. Protein phosphatase 2A: a target for anticancer therapy. Lancet Oncol. 2013; 14: e229–238. 10.1016/S1470-2045(12)70558-2 23639323 PMC3913484

[46] Ceulemans H, Bollen M. Functional Diversity of Protein Phosphatase-1, a Cellular Economizer and Reset Button. Physiol Rev. 2004; 84: 1–39. 10.1152/physrev.00013.2003 14715909

[47] Jaumot M, Hancock JF. Protein phosphatases 1 and 2A promote Raf-1 activation by regulating 14-3-3 interactions. Oncogene. 2001; 20: 3949–3958. 10.1038/sj.onc.1204526 11494123

[48] Chuang M-J, Wu S-T, Tang S-H, Lai X-M, Lai H-C, Hsu K-H, et al The HDAC Inhibitor LBH589 Induces ERK-Dependent Prometaphase Arrest in Prostate Cancer via HDAC6 Inactivation and Down-Regulation. PLoS One. 2013; 8: e73401 10.1371/journal.pone.0073401 24023871 PMC3762759

[49] Valjent E, Pascoli V, Svenningsson P, Paul S, Enslen H, Corvol J-C, et al Regulation of a protein phosphatase cascade allows convergent dopamine and glutamate signals to activate ERK in the striatum. Proc Natl Acad Sci U S A. 2005; 102: 491–496. 10.1073/pnas.0408305102 15608059 PMC544317

